# An Atypical Root Canal Configuration of Maxillary Second Molar: A Case Report 

**DOI:** 10.30476/dentjods.2025.107040.2714

**Published:** 2026-09-01

**Authors:** Ali Rezaei Nejad, Yasamin Ghahramani, Fahime Alimardani, Hasti Hosseini

**Affiliations:** 1 Postgraduate Student, Dept. of Endodontics, School of Dentistry, Shiraz University of Medical Sciences, Shiraz, Iran.; 2 Dept. of Endodontics, School of Dentistry, Shiraz University of Medical Sciences, Shiraz, Iran.; 3 Undergraduate Student, Dept. of Endodontics, School of Dentistry, Shiraz University of Medical Sciences, Shiraz, Iran.

**Keywords:** Maxillary Second Molar, Root Canal Therapy, Complex Anatomy, Root Canal Morphology

## Abstract

The main goals of root canal therapy are to relieve pain, eliminate infections from the root canal system, and prevent future infection. The maxillary second molars typically present with three roots and contain three to four canals; however, anatomical variations frequently observed. Failure to identify these variations can complicate the procedure, emphasizing the importance of precise radiographic assessment. Cone beam computed tomography (CBCT) has demonstrated superior effectiveness compared to conventional radiography in assessing root canal morphology. This paper reports a maxillary second molar with unusual root morphology having two palatal roots. CBCT confirmed this diagnosis.

## Introduction

Root canal therapy is primarily performed to alleviate pain, eliminate infections within the root canal, and prevent their recurrence [ 1 ]. The achievement of successful root canal treatment hinges on several critical procedural steps, which include cleaning, shaping, and obtaining three-dimensional obturation of the root canal system. These steps require not only technical proficiency but also a deep conceptual understanding of the complex anatomy of the root canal system [ 2 ]. A significant number of challenges encountered during root canal treatment can be directly caused by a limited or inadequate understanding of tooth root morphology, which can ultimately lead to suboptimal treatment outcomes [ 1 ]. Therefore, a thorough appreciation of the root canal anatomy is paramount to ensuring the effectiveness of the procedure. Neglecting the anatomical diversity found in maxillary molars especially the second molars, might result in significant procedural failures. The maxillary second molars typically comprise three roots, each containing either three or four canals as two canals located in the mesiobuccal (MB) root, one canal in the distobuccal (DB) root, and one canal in the palatal root [ 3 ]. Various normal anatomical variations have been documented in the literature. Shojaeian *et al* . [ 4 ] reported a rare anatomical variation of a maxillary second molar with two palatal canals, successfully diagnosed and treated. Furthermore, Badole *et al* . [ 1 ] described a rare case of a maxillary right second molar featuring two palatal canals and a total of four root canals.

The role of precise radiographic techniques cannot be overstated in this context, as these methods provide critical insights into the root canal system, allowing for proper diagnosis, treatment planning, and procedural success. Radiographic imaging, when interpreted correctly, is essential for identifying root canal morphology and can significantly influence treatment outcomes. Pre-operative radiographs, especially those taken from multiple angles, are invaluable for outlining and evaluating the root canal anatomy, as they help clinicians visualize the precise configuration and any potential anatomical variations that may affect the treatment plan [ 1 ].

Among the various imaging technologies used to assess root canal morphology, cone beam computed tomography (CBCT) stands out for its ability to provide high-resolution, three-dimensional imaging. CBCT has demonstrated superior diagnostic accuracy compared to conventional radiography, offering enhanced visualization of the internal anatomy of the root canal system. This advanced imaging modality allows clinicians to obtain detailed, three-dimensional views of the tooth’s root structure, enabling more precise treatment planning and improving overall clinical outcomes. The ability to detect and assess root canal anatomy in such detail plays a crucial role in optimizing the efficiency of root canal therapy, reducing procedural risks, and ultimately contributing to more successful and predictable results [ 5 ]. 

## Case Presentation 

**Figure 1:**
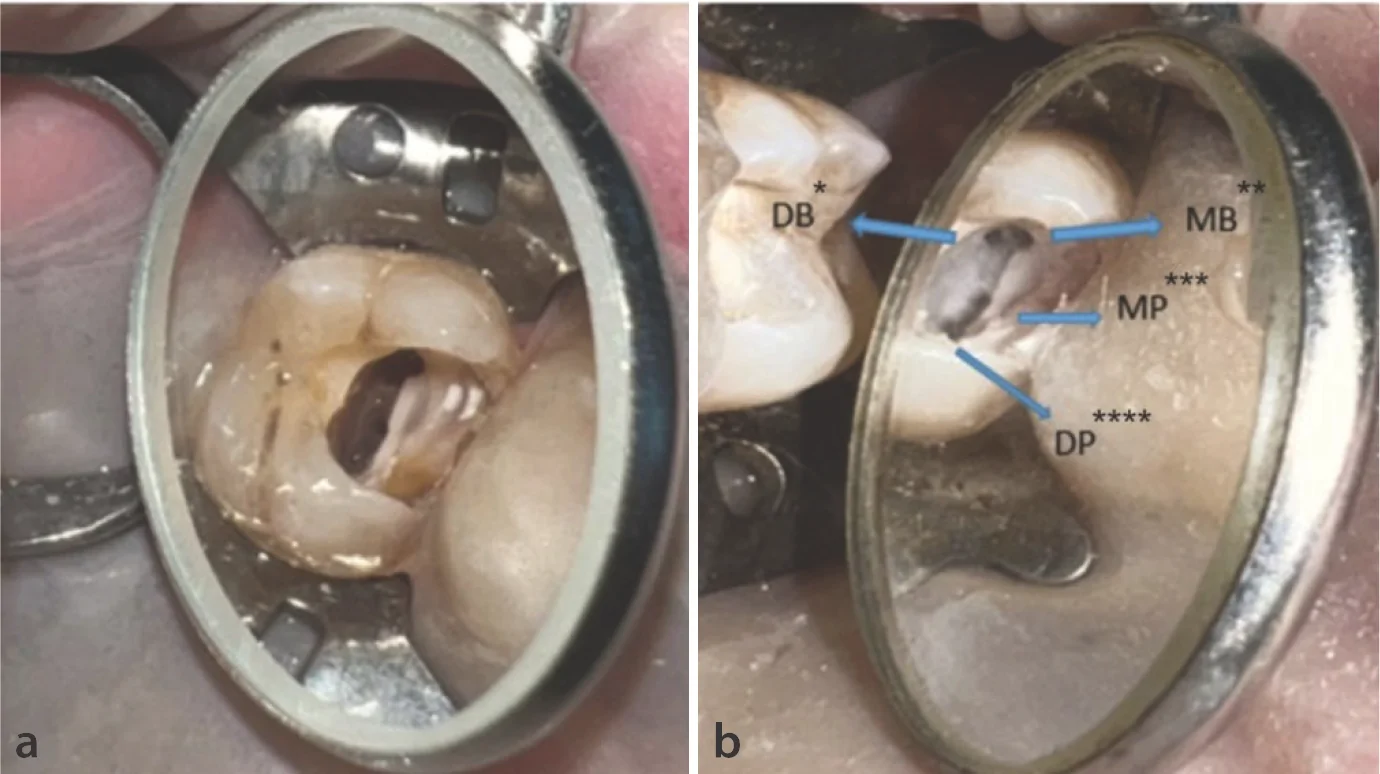
Location of orifices (* DB: distobuccal, ** MB: mesiobuccal, *** MP: mesiopalatal, **** DP: distopalatal)

**Figure 2:**
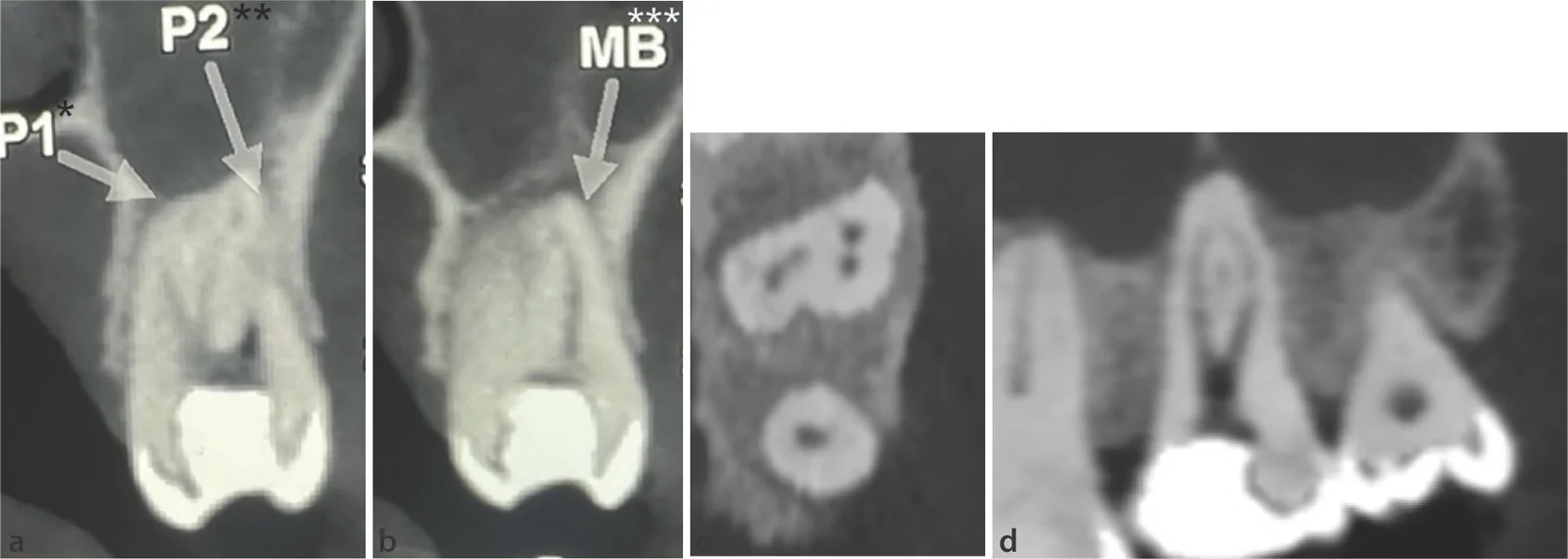
CBCT images **a:** Coronal view, **b:** Coronal view, **c:** Axial view, **d:** Sagittal view (*P1: palatal canal 1, **P2: palatal canal 2, ***MB: mesiobuccal)

**Figure 3:**
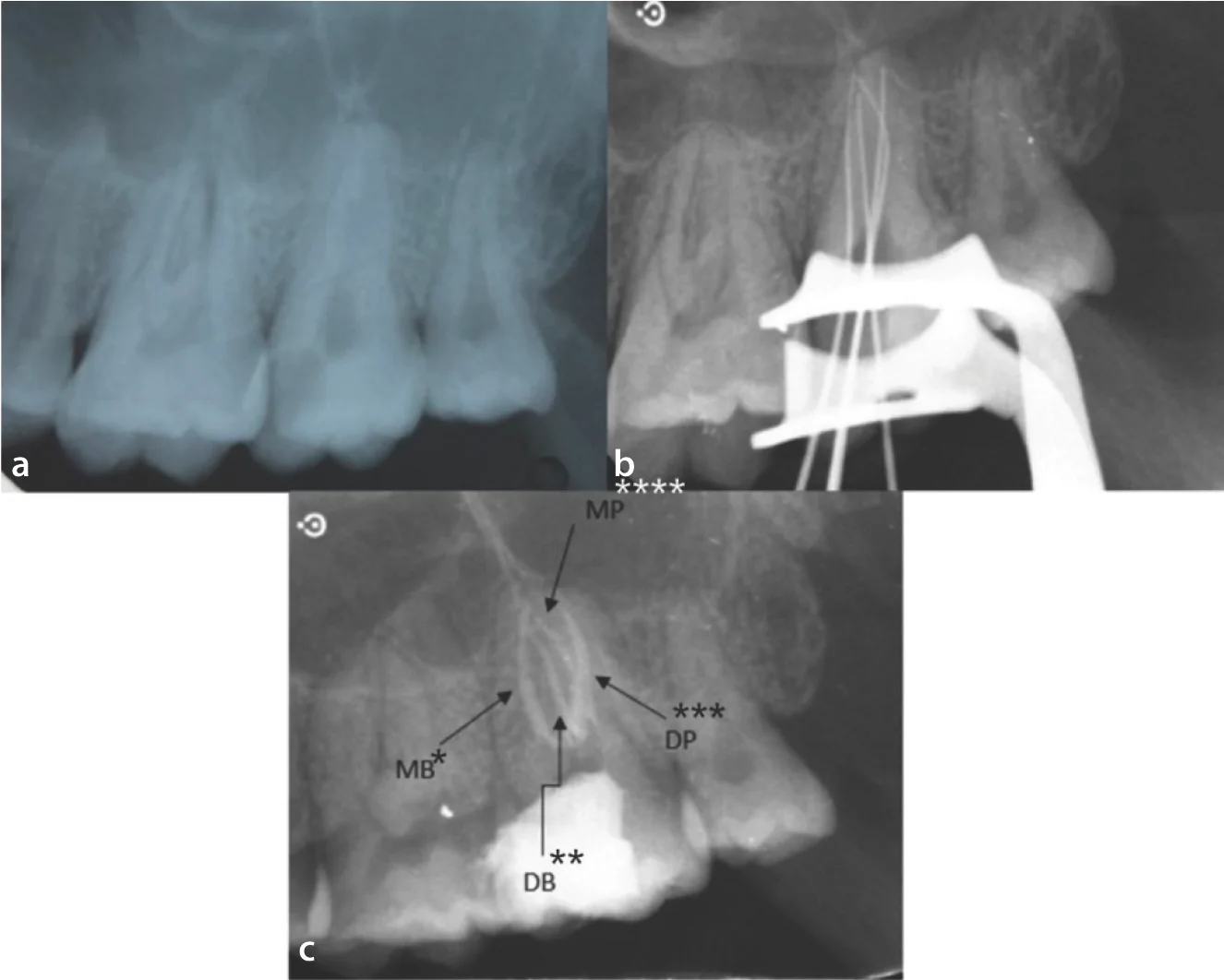
**a:** Pre operative radiography, **b:** Initial files for working length, **c:** Post operative radiography showing 4 canals of this case as (* MB: mesiobuccal, **DB: distobuccal, ***DP: distopalatal, ****MP: mesiopalatal)

 A 33-year-old male patient was referred to the Endodontics Department at Shiraz University of Medical Sciences for the management of persistent pain in his maxillary left second molar, which had been ongoing for three days. A radiographic evaluation of tooth #15 revealed the presence of mesial caries approaching the pulp chamber, with no evident of periapical pathology. Clinical test demonstrated a severe response to cold test and a positive result on electric pulp test. Based on clinical and radiographic findings, the tooth was diagnosed with symptomatic irreversible pulpitis, and the apical tissues were deemed normal. Following administration of appropriate local anesthesia and placement of a rubber dam for isolation, access to the pulp chamber was prepared using a fissure diamond bur. Upon performing a detailed clinical examination of the pulp chamber floor, four distinct root canal orifices were identified- two situated buccally and two located palatally. However, the arrangement of these orifices was atypical. Notably, an additional orifice was found between the MB and palatal canals, which was positioned closer to the palatal canal. This anatomical variation led to the conclusion that the root canal located in the mesiopalatal (MP) corner should be classified as the MP canal, rather than the traditionally expected second mesiobuccal canal (MB2). This finding highlights the importance of thorough clinical evaluation and awareness of potential anatomical deviations in the root canal system (Figure 1). CBCT was employed to evaluate the complexity of the root canal anatomy and to detect any potential accessory canals or anatomical features that could complicate the treatment process. CBCT scans of tooth #15 were conducted to provide a detailed view of the anatomical structures. A series of CBCT images and 3D reconstruction images were obtained, which revealed that tooth #15 contained four root canals: MB canal, MP canal, DB canal, and distopalatal canal, all within two roots- buccal and palatal (Figure 2a). The orifice located at the MP corner was confirmed to be the MP canal (Figure 2a). Furthermore, the CBCT images revealed that the MB root and DB root were fused, an anatomical variation that was visualized in the imaging (Figure 2d). This detailed imaging confirmed the presence of intricate canal anatomy, underlining the importance of advanced imaging techniques in assessing complex root canal systems. During the subsequent appointment, the working length of each root canal was initially determined using an apex locator (Root ZX, Morita, Tokyo, Japan), and verified through digital intraoral periapical radiographs (Figure 3b). The root canals were cleaned and shaped utilizing nickel-titanium (NiTi) rotary ProTaper files (Dentsply Maillefer, Ballaigues, Switzerland) in accordance with the crown-down technique, as described in the literature [ 9 ]. The apical preparation of the buccal canals was enlarged to a ProTaper size F2. Throughout the instrumentation, the canals were frequently irrigated with 5.25% sodium hypochlorite (Prime Dental Products, Thane, India) to ensure effective disinfection. This was followed by the application of 17% ethylenediaminetetraacetic acid (EDTA) (Smear Clean®, Nippon Shika Yakuhin Co., Ltd.) to remove the smear layer, ensuring optimal cleaning of the canal walls. After irrigation, the canals were dried using sterile paper points to prepare for obturation. Finally, the root canals were obturated with AH-26 sealer (Dentsply DeTrey, Konstanz, Germany) in combination with ProTaper gutta-percha cones, employing lateral condensation ensuring a three-dimensional seal of the entire root canal system. 

This comprehensive approach ensured the thorough cleaning, shaping, and sealing of the root canal system, following established protocols for effective root canal therapy. 

Prior to data collection, informed consent was obtained from the patient for the use of their medical information in this study. All data were handled confidentially and in accordance with ethical principles.

## Discussion

The internal morphology of human teeth is inherently complex and shows considerable variability. Maxillary second molar, like other teeth, exhibit various anatomical differences. Typically, these molars have three distinct roots. However, in endodontic procedures, variations in root canal morphology present significant challenges that necessitate a profound understanding of the anatomical diversities. These anatomical variations underscore the importance of utilizing advanced imaging techniques and classification systems, as they increase the potential for complications during both diagnosis and treatment. The majority of maxillary second molar typically present with three roots; anomalies such as additional canals and root fusions are not uncommon and can complicate treatment [ 6 - 8 ]. The presence of more than one palatal canal has been reported in maxillary first and second molars; however, its prevalence is higher in the second molars. For accurate diagnosis and effective treatment planning, a comprehensive understanding of these morphological complexities is essential [ 6 - 8 ].

A retrospective study conducted by Peikoff *et al* . [ 9 ] identified six distinct types of maxillary second molars. The results showed that 56% of the molars had three distinct roots and canals, 22.7% had three roots but four canals (including two MB canals), 9% exhibited three roots with fused MB and DB canals, 6.9% had two separate roots with corresponding canals, 3.1% had a single root with one canal, and 1.4% had four roots or canals, including two palatal canals. Furthermore, Nabaviadeh *et al* . [ 10 ] described a maxillary second molar that featured two palatal canals converging into a single apex. While clinical guidelines and standardized definitions exist for identifying root canal orifice locations- guidelines that aid in determining the position of the pulp chamber as well as the number and precise locations of the root canals- it remains crucial to meticulously evaluate any indications of additional root canals in such cases [ 11 ]. In the present case, the second palatal orifice was located between the MB and palatal orifices within the pulp chamber. However, as the canal extended toward the root, it deviated toward the buccal root. The buccal roots were fused, and the palatal root was found to be in close proximity to the buccal root, as evidenced by the CT scan. According to the mentioned CBCT slice (Figure 3), the MP canal initially moves toward the palatal side and joins the palatal canal, forming a single merged pathway. Subsequently, as it progresses apically, the canal separated again and changed its direction toward the buccal root. Additionally, in this slice, it appears that the apices of the MP and MB canals are distinct.

 In more complex cases, CBCT serves as a highly effective imaging modality that significantly enhances the precision of diagnosis and treatment planning. CBC-T enables clinicians to make more informed decisions by providing detailed and accurate three-dimensional visualization of root canal anatomy [ 12 ]. One study demonstrated that after reviewing CBCT images, 30% of clinicians revised their initial treatment plans [ 13 ]. As seen in this particular case, there exists a wide range of possible anatomical variations. In this context, CBCT plays an indispensable role by offering precise and comprehensive imaging, thereby facilitating the identification of such variations and enabling more effective treatment strategies. 

## Conclusion

The internal configuration of the maxillary second molar is complex, with significant anatomical variability. Advanced imaging techniques like CBCT provide critical insights into these variations, improving diagnosis, treatment planning, and clinical outcomes. A proper understanding and interpretation of these anatomical differences are crucial for successful root canal treatment.
